# A cross-sectional survey on respiratory disease in a cohort of Irish pig farms

**DOI:** 10.1186/s13620-020-00176-w

**Published:** 2020-11-21

**Authors:** Maria Rodrigues da Costa, Rose Mary Fitzgerald, Edgar Garcia Manzanilla, Helen O’Shea, John Moriarty, Máire C. McElroy, Finola Catherine Leonard

**Affiliations:** 1 Pig Development Department, Teagasc - Animal & Grassland Research and Innovation Centre, Moorepark, Fermoy, P61 C996 Co. Cork, Ireland; 2 Departament de Ciencia Animal i dels Aliments, Facultat de Veterinaria, Universitat Autònoma de Barcelona, Bellaterra, 08193 Barcelona, Spain; 3 School of Veterinary Medicine, University College Dublin, Dublin 4, D04 V1W8 Belfield, Ireland; 4 Present address: Epidemiology Research Unit, Department of Veterinary and Animal Science, Northern Faculty, Scotland’s Rural College (SRUC), An Lòchran, 10 Inverness Campus, IV2 5NA Inverness, Scotland, UK; 5 Bio-Explore, Department of Biological Sciences, Cork Institute of Technology, Rossa Avenue, Bishopstown, T12 P928 Cork, Ireland; 6 Central Veterinary Research laboratory, Department of Agriculture, Food & Marine Laboratories, Celbridge W23VW2C Co. Kildare, Ireland

**Keywords:** *Actinobacillus pleuropneumoniae*, Influenza A virus, Lung scoring, *Mycoplasma hyopneumoniae*, Pluck lesions, Porcine reproductive and respiratory syndrome virus, Porcine respiratory disease complex, Seroprevalence

## Abstract

**Background:**

Respiratory disease is one of the most important factors impacting pig production worldwide. There is no available information on the prevalence of key pathogens implicated in Irish pig production. The objective of this study was to describe the prevalence of pleurisy, pneumonia, lung abscesses, pericarditis and liver milk spots in finisher pigs of a cohort of Irish pig farms, and to describe the seroprevalence of: influenza A virus (IAV), porcine reproductive and respiratory syndrome virus (PRRSv), *Mycoplasma hyopneumoniae* (Mhyo) and *Actinobacillus pleuropneumoniae* (APP).

**Results:**

In brief, 56 farrow-to-finish farms (29% of the Irish breeding herd) were enrolled in the study in 2017. Data on lungs, heart, and liver lesions were assessed for each farm at slaughter. An average of 417 (range 129–1154) plucks per farm were assessed for pleurisy, pneumonia, lung abscesses, pericarditis, and liver milk spots. Blood samples from 32 finisher pigs were collected at slaughter for each farm. The observed prevalence of pleurisy and pneumonia was one of the lowest reported in similar studies in Europe (13 and 11% estimated average within farm, respectively). Pleurisy lesions were mostly moderate and severe. Pneumonia lesions affected a low level of lung surface (5.8%). Prevalence of pericarditis was mid-high (8%) and the prevalence of liver milk spots was high, with an average of 29% of the livers affected. For serology, 78.6% of the farms were positive for IAV, 50% were positive for PRRSv, 71.4% were positive for Mhyo, and 98.2% were positive for APP. Influenza virus was the main pathogen associated with pleurisy (*P* < 0.001) and Mhyo was the main pathogen associated with pneumonia (*P* < 0.001) and pericarditis (*P* = 0.024).

**Conclusions:**

Farms affected with pleurisy had moderate to severe lesions. Farms affected with pneumonia had mild lesions, which could be the effect of the generalised use of Mhyo vaccination in piglets. The seroprevalence of IAV, PRRSv, Mhyo and APP in the present study sample is similar to or lower than in other European countries. Further research on the PRRSv and APP strains circulating in Ireland is necessary to support the design of national or regional control plans.

## Background

Respiratory disease is one of the most important factors impacting pig production worldwide. Agents contributing to the porcine respiratory disease complex include viruses and bacteria such as Influenza A virus (IAV), Porcine reproductive and respiratory syndrome virus (PRRSv), *Mycoplasma hyopneumoniae* (Mhyo) and *Actinobacillus pleuropneumoniae* (APP) [1]. The relative importance of each one of these pathogens varies between countries and regions due to factors such as structure of farms, husbandry practices or climate. In Ireland, there is no baseline information available on the prevalence of respiratory pathogens and disease in pig farms. However, the future implementation of control and eradication programmes requires the characterisation of the national herd health status [2]. This national characterisation is also important for individual farms, given their susceptibility to new outbreaks when the regional disease prevalence is high.

Veterinary practitioners carry out regular diagnostics to monitor the health status of pig farms and the efficacy of disease control measures e.g. vaccination [3]. Slaughterhouse checks, including lung scoring and the recording of other lesions e.g. pericarditis and liver milk spots (caused by *Ascaris suum*) are inexpensive monitoring tools, allowing the collection of data from several farms at one time point [4]. Serology in finisher pigs at slaughter also allows estimation of the prevalence of exposure to pathogens or the efficacy of vaccination on farm [5, 6]. Combining slaughterhouse checks and serology with information on vaccination protocols is a useful approach to study the health status of farms in terms of respiratory disease.

Thus, the aims of this study were (1) to describe the prevalence of pleurisy, pneumonia, lung abscesses, pericarditis and liver milk spots in finisher pigs of a cohort of Irish farrow-to-finish pig farms, (2) to describe the seroprevalence of four main pathogens related to respiratory disease: IAV, PRRSv, Mhyo and APP in those farms, and (3) to analyse the associations between the average within-farm prevalence of pluck lesions and farm health status for the pathogens described by serology.

## Methods

Data on lung lesions, the presence of pericarditis and liver milk spots, and also blood samples were obtained during visits to eight slaughterhouses (seven in the Republic of Ireland and one in Northern Ireland, UK) from November 2017 to April 2018, targeting 56 Irish farrow-to-finish pig farms. This cohort of farms was part of a larger study selected from the Teagasc e-Profit Monitor (ePM) clients. The Teagasc ePM is a herd monitoring system (production performance and economic data) available on a voluntary basis to all the farmers in the Republic of Ireland. In 2017, it included 107 pig herds, representing over 77,000 sows or 52% of the national commercial sow herd [7]. Participation in the cross-sectional study was offered to all the farrow-to-finish pig farmers providing data to the ePM, and 56 farms participated voluntarily (cohort in study). Farms were recruited through the Teagasc advisory service and represented 29.2% of the national commercial sow herd.

At least two batches per farm were assessed with blood samples being harvested from the first batch. A batch was defined as all the finisher pigs from a given farm killed in a slaughterhouse on the same day. Vaccination data were obtained via phone calls to farmers and corresponding private veterinary practitioners during the same period. Additionally, farmers and veterinarians were asked if there were any changes in the vaccination scheme in the previous year. Farm characteristics for the participating farms were retrieved from the Teagasc ePM.

All the farmers participating in this study provided individual signed consent to the use of the farm data collected, and to the retrieval of their production data from the Teagasc ePM, according to Teagasc´s internal data protection regulation.

### Blood sampling and pluck examinations at slaughter

In the slaughterhouse, blood was collected from a total of 32 randomly selected pigs per farm at sticking (exsanguination). The number of pigs to be sampled was chosen to detect viral or bacterial infections with a minimum within-herd prevalence of 10% (α = 0.05). Samples were transported for analysis to the Blood Testing Laboratory of the Department of Agriculture, Food and the Marine (Cork, Ireland). Blood was allowed to clot at room temperature, serum was separated, aliquoted and frozen at -80ºC until required for testing. For analysis, 16 randomly selected samples per farm for PRRSv and Mhyo, and all 32 samples per farm for IAV and APP were tested, based on power calculation for detection of viral and bacterial infections. The number of pig samples processed for the detection of IAV and APP (*n* = 32) allowed a minimum within-herd prevalence of 10% (α = 0.05), while the number of samples processed for the detection of PRRSv and Mhyo (*n* = 16) allowed a minimum within-herd prevalence of 18% (α = 0.05). The processing of half of the samples for the former two pathogens was based on its seroconversion dynamics. Previous studies suggest that, once a farm is infected, the disease prevalence in finisher pigs tends to 100%, particularly in farrow-to-finish farms [8, 9].

Pluck (lungs, heart, and liver) examinations were all carried out by the same trained veterinarian. For each pig, lung lobes were scored for pneumonia lesions according to the method described by Madec and Derrien [10], with the overall surface affected averaged accounting for lobe weights [11]. The variables prevalence of pneumonia (%) and average surface of pneumonic lungs (%), hereinafter called (lung) surface with pneumonia (%), were used for statistical analysis. Pleurisy was scored in the dorsocaudal lobes using a modified version of the Slaughterhouse Pleurisy Evaluation System (SPES), which was developed by Dottori et al., [12] and described by Merialdi et al., [13]. The scores were 0 (no pleurisy), 2 (focal lesions in one lobe), 3 (bilateral adhesions or unilateral lesions affecting more than 1/3 of the diaphragmatic lobe), and 4 (extensive lesions affecting more than 1/3 of both diaphragmatic lobes). The prevalence of pleurisy (lesions with SPES ≥ 2) and the prevalence of scores 3 and 4 (prevalence of moderate or severe dorsocaudal pleurisy) were used for statistical analysis. Cranial pleurisy (adhesions between lobes, on the surface of the apical and cardiac lobe, and/or adhesions between the lung and the heart), which corresponded to score 1 of the original SPES, and scars (healing indicative of pneumonic lesions which developed earlier in the pig’s life) were recorded as absent or present and used in the analysis. In summary, all pleurisy-related variables included pleurisy, moderate and severe pleurisy and cranial pleurisy, while pneumonia-related variables were included pneumonia, lung surface with pneumonia, and scars. Additionally, lung abscesses (presence of one or more abscesses in the lung), pericarditis (defined as expansion of the pericardial cavity with inflammatory exudate [14]), and liver milk spots (presence of white spots in the liver indicative of transhepatic migration of the larvae of *Ascaris suum* [15]) were also recorded as absent or present. In slower speed lines, every pig in the batch was scored while in the fastest speed line (max. 600 per hour), every other pluck was assessed. The pluck examinations here described were recorded using the Ceva Lung Program app (CEVA Santé Animale, Libourne, France).

### Serology

Seroprevalence of antibodies against IAV, PRRSv, Mhyo and APP were determined using the following IDEXX ELISA kits (Hoofddorp, The Netherlands), respectively: Influenza A Ab Test (95.3% sensitivity, 99.6% specificity for swine sera), PRRS X3 Ab Test (for the detection of both the European and North American genotypes with 98.8% sensitivity, and 99.9% specificity), HerdChek *Mycoplasma hyopneumoniae* Antibody Test (89.4% sensitivity, 99.67% specificity), APP-ApxIV Ab Test (97.8% sensitivity, 100% specificity). Following the manufacturers’ recommendations, each pig was considered positive to: IAV if their S/N value (sample to negative ratio value) for IAV was less than 0.6, PRRSv if their S/P value (sample to positive ratio value) was greater or equal to 0.4, Mhyo if their S/P values were greater than 0.4, and to APP if their S/P values were greater or equal to 0.5. ELISA results were transcribed into two variables per infectious pathogen: farm status (farms were considered positive if at least one animal tested positive by ELISA test) and within farm prevalence (number of pigs positive divided by the total number of pigs tested per farm).

### Vaccination

The main vaccination protocols on farm were recorded, with special focus on vaccination for IAV, PRRSv, Mhyo and APP in sows and in piglets, as present or absent.

### Data input, management and analysis

All data were entered and collated into a Microsoft® Excel 365 spreadsheet. Descriptive statistical analysis and data visualisation were completed using R version 3.4.4 [16] and R package ggplot2 version 3.3.0 [17]. Pluck lesions, vaccination, and serology (farm status, within-farm prevalence) were characterised and described. Herd-level prevalence was calculated as the number of positive herds (farm status positive) divided by the total number of herds (*n* = 56). Herd-level true prevalence was estimated from apparent prevalence as described by Rogan & Gladen (1978) [18] and reported in Thrusfield (2007) [19]. The results for pluck lesions are presented as average within-farm prevalence.

Variables were checked for normality using graphic examination and Kolmogorov-Smirnov test. Productive performance for the cohort of farm was compared to the general population using ANOVA with an alpha level for determination of significance of 0.05. The associations between average within-farm prevalence for pluck lesions and farm status was explored using SAS 9.4 (SAS Institute, Carey, US). Univariate analysis of the associations of pluck lesions and serology was carried out using Mann-Whitney U test. Multivariable analysis was carried out using generalized linear models with manual stepwise selection of variables with *P* = 0.10 and *P* = 0.05 as inclusion and retention criteria.

## Results

### Farm performance and herd characteristics

A total of 56 farrow-to-finish farms participated in this study. A summary of the farm characteristics and their performance in comparison to the Teagasc ePM population for the year 2017 is shown in Table 1. No statistical differences were found for any parameter between the two groups of farms (*P* > 0.05). The average live weight at which pigs were sent to slaughter (111 ± 4.9 kg) depended on the sale target defined by each farmer which also considered slaughterhouse preferences.

**Table 1:** Description of the farm characteristics and production performance of a cohort of 56 Irish farrow-to-finish pig farms (study farms) and 2017 data from all herds in the Teagasc e-Profit Monitor system (population; *n* = 107)

Farm characteristics and performance	Teagasc e-Profit Monitor^**a**^	Study farms	
	mean ± SD	mean ± SD	Median (range)
**Average herd size (number of sows)**	728 ± 487.2	789 ± 564.1	659 (109-2498)
**No. pigs produced per sow per year**	26.4 ± 2.31	26.7 ± 2.23	26.5 (21.8-32.0)
**Weaner mortality, %**	2.8 ± 1.63	2.8 ± 1.61	2.7 (0.5-8.9)
**Finisher mortality, %**	2.2 ± 1.19	2.0 ± 0.76	1.8 (0.9-4.1)
**Average daily gain, g/day**	711 ± 62.8	726 ± 62.6	725 (538-903)
**Feed conversion ratio**	2.37 ± 0.335	2.38 ± 0.105	2.38 (2.21-2.68)
**Age at sale, day**	175 ± 12.9	174 ± 11.8	172 (148-208)

^a^National herd monitoring system for production performance and economic data which is available on a voluntary basis

### Pluck lesions

**Fig. 1:**
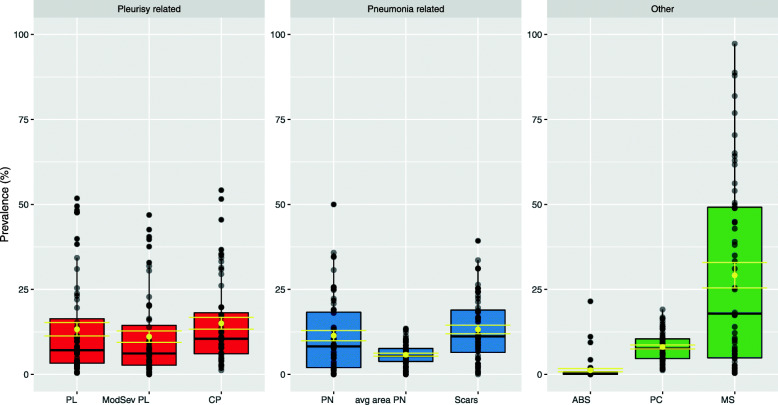
Prevalence (%) of lung lesions, pericarditis and liver milk spots in finisher pigs of a cohort of 56 Irish farrow-to-finish farms from November 2017 to April 2018 (mean ± SE in yellow). A total of 23,372 plucks were assessed, with an average of 417 ± 204.1 plucks assessed per farm (range 129 - 1154). Legend: PL – dorsocaudal pleurisy; ModSev PL – Moderate and severe dorsocaudal pleurisy; CP – Cranial Pleurisy; PN – Pneumonia; Avg area PN – average lung surface affected with pneumonia; ABS – lung abscesses; PC – pericarditis; MS – Liver milk spots. Scars correspond to the healing indicative of pneumonic lesions

The prevalence of lung lesions, pericarditis and liver milk spots per farm recorded at slaughter is presented in Fig. 1. A total of 23,372 plucks were assessed at slaughter. Data is presented as mean ± SD across all farms. On average, each batch had 163 ± 55.5 plucks assessed and 2.6 batches were assessed per farm, with a minimum of two batches assessed and a maximum of five batches assessed. An average of 417 ± 204.1 plucks were assessed per farm (range 129–1154).

The prevalence of pleurisy was 13 ± 14.7%. Out of these lesions, 73% of these were moderate and severe lesions (SPES scores 3 and 4). The prevalence of pneumonia on farm was (11 ± 11.2%), with an average surface affected of around 5.8%. Each farm had an average of 13 ± 9.4% of scarred lungs. The average within-farm prevalence of lung abscesses, pericarditis, and liver milk spots was 1.2 ± 3.38%; 8.1 ± 4.39% and 29.2 ± 28.04%, respectively.

### Vaccination and farm serology results

A total of 39.3 and 42.9% of the farms were vaccinating sows for IAV and PRRSv, respectively. Additionally, one farm reported also vaccinating piglets for IAV, and five farms were also vaccinating piglets for PRRSv. A total of 73.2% of the farms were vaccinating piglets for Mhyo. Of the farms vaccinating piglets, 39% administered a double shot. APP vaccination was only used in five farms (8.9%), all of which vaccinated weaner pigs with one farm also vaccinating sows.

Approximately 94.6% of the farms vaccinated piglets for PCV2 (Porcine circovirus type 2), 80.4% vaccinated sows and gilts for *Escherichia coli*, 17.9% for *Clostridium* spp., and 7.1% reported vaccinating piglets for atrophic rhinitis. All farms vaccinated their sows for porcine parvovirus and *Erysipelothrix rhusiopathiae*, and none vaccinated for *Glaesserella parasuis*.

**Fig. 2:**
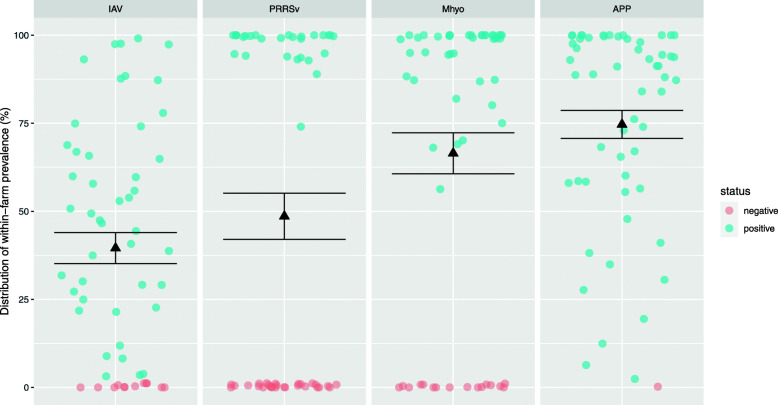
Distribution of within-farm prevalence (%) of influenza A virus (IAV), porcine reproductive and respiratory virus (PRRSv), *Mycoplasma hyopneumoniae* (Mhyo), and *Actinobacillus pleuropneumoniae* (APP) in finisher pigs of 56 Irish farrow-to-finish farms (mean ± SE in black). Farms were considered positive if one pig was positive

The results of the serological survey showed that the apparent herd-level prevalence in the study sample was 78.6% for IAV, 50.0% for PRRSv, 71.4% for Mhyo, and 98.2% for APP. True herd-level prevalence was estimated to be 82% [95% CI 71.0–93.7%], 51% [95% CI 37.3–63.8%], 80% [95% CI 66.6–93.1%] and 100% [95% CI 96.9–100%] for IAV, PRRSv, Mhyo and APP, respectively. In positive farms, the within-farm prevalence for each pathogen was (mean ± SD): 50.3 ± 28.88% for IAV, 97.2 ± 5.44% for PRRSv, 93.0 ± 11.31% for Mhyo, and 76.0 ± 28.15% for APP. The distribution of within-farm prevalence for the different pathogens is presented in Fig. 2.

### Associations between farm health status and the within herd average prevalence of pluck lesions

Table 2 presents the univariate and multivariate analysis for all the pluck lesions depending on serology for IAV, PRRSv, Mhyo; APP could not be included in the analysis because almost all farms were positive. All variables related to pleurisy and pneumonia were associated with the three studied pathogens in the univariate analysis. In the multivariable analysis, higher level of dorsocaudal pleurisy, moderate and severe pleurisy and cranial pleurisy were associated with positive status for IAV (*P* < 0.001 in all cases). Pneumonia, surface affected by pneumonia and scars were higher for farms positive for Mhyo (*P* ≤ 0.001 in all cases). IAV was also associated with higher levels of pneumonia (*P* = 0.023) and scars (*P* = 0.008). Abscesses tended to be associated with IAV (*P* = 0.061) and pericarditis was associated with Mhyo (*P* = 0.024). Liver milk spots were not associated with any of the pathogens.

**Table 2:** Univariate and multivariable analysis of average within herd prevalence (%) of pluck lesions and lung surface affected with pneumonia depending on farm serology for influenza A virus (IAV), porcine respiratory and reproductive syndrome virus (PRRSv) and *Mycoplasma hyopneumoniae* (Mhyo)^a,b^

	IAV	PRRSv	Mhyo
**Dorsocaudal Pleurisy**	Pos: 9.8 (0.9, 51.8) Neg: 2.4 (0.4, 47.7)	Pos: 10.2 (2.8, 51.8) Neg: 4.0 (0.4, 49.5)	Pos: 8.2 (2.0, 51.8) Neg: 2.9 (0.4, 47.7)
Univariate	*P* < 0.001	*P* = 0.008	*P* = 0.009
Multivariable	*P* < 0.001	-	-
**Mod Sev dorsocaudal pleurisy**	Pos: 7.5 (0.7, 46.9) Neg: 1.7 (0.0, 42.6)	Pos: 8.7 (1.8, 46.9) Neg: 3.8 (0.0, 42.6)	Pos: 6.8 (0.5, 46.9) Neg: 2.6 (0.0, 42.6)
Univariate	*P <* 0.001	*P* = 0.006	*P* = 0.025
Multivariable	*P <* 0.001	-	-
**Cranial pleurisy**	Pos: 12.4 (1.9, 54.2) Neg: 4.8 (1.2, 35.1)	Pos: 12.4 (4.2, 54.2) Neg: 7.1 (1.2, 51.6)	Pos: 10.9 (2.5, 54.2) Neg: 5.8 (1.2, 35.1)
Univariate	*P* = 0.001	*P* = 0.007	*P* = 0.032
Multivariable	*P <* 0.001	-	-
**Pneumonia**	Pos: 9.5 (0.7, 50.0) Neg: 1.5 (0.0, 18.9)	Pos: 10.3 (1.3, 34.7) Neg: 2.6 (0.0, 50.0)	Pos: 11.0 (2.0, 50.0) Neg: 1.3 (0.0, 30.7)
Univariate	*P* = 0.003	*P* = 0.035	*P <* 0.001
Multivariable	*P* = 0.023	-	*P <* 0.001
**Avg pneumonia surface**^**c**^	Pos: 6.0 (1.0, 13.5) Neg: 2.7 (0.0, 9.5)	Pos: 6.4 (2.2, 13.5) Neg: 4.6 (0.0, 10.6)	Pos: 6.5 (2.0, 13.5) Neg: 2.3 (0.0, 6.6)
Univariate	*P* = 0.002	*P* = 0.014	*P* < 0.001
Multivariable	-	-	*P* < 0.001
**Scars**	Pos: 12.5 (1.9, 39.3) Neg: 2.3 (0.0, 23.1)	Pos: 17.5 (1.4, 39.3) Neg: 8.0 (0.0, 24.3)	Pos: 15.9 (3.1, 39.3) Neg: 2.5 (0.0, 13.2)
Univariate	*P* < 0.001	*P* < 0.001	*P* < 0.001
Multivariable	*P* = 0.008	-	*P* < 0.001
**Abscesses**	Pos: 0.5 (0.0, 11.1) Neg: 0.1 (0.0, 21.5)	Pos: 0.3 (0.0, 4.3) Neg: 0.3 (0.0, 21.5)	Pos: 0.5 (0.0, 11.1) Neg: 0.2 (0.0, 21.5)
Univariate	*P* = 0.073	*P* = 0.842	*P* = 0.194
Multivariable	*P* = 0.061	-	-
**Pericarditis**	Pos: 8.0 (1.2, 19.1) Neg: 6.1 (1.5, 14.8)	Pos: 8.0 (2.3, 16.4) Neg: 7.4 (1.2, 19.1)	Pos.: 8.2 (1.5, 19.1) Neg.: 5.5 (1.2, 15.6)
Univariate	*P* = 0.251	*P* = 0.561	*P* = 0.018
Multivariable	-	-	*P* = 0.024
**Liver milk spots**	Pos: 27.1 (0.5, 97.3) Neg: 12.4 (0.4, 88.8)	Pos: 24.4 (0.5, 87.9) Neg: 17.8 (0.4, 97.3)	Pos.: 15.7 (0.4, 88.8) Neg.: 25.3 (0.4, 97.3)
Univariate	*P* = 0.374	*P* = 0.838	*P* = 0.899
Multivariable	-	-	-

^a^Univariate analysis was carried using Mann-Whitney test and data is presented as median (range) for positive (pos) and negative (neg) farms. Multivariable analysis was carried out using generalized linear models with forward selection with a P for inclusion of 0.10 and a *P* for retention of 0.05

^b^Results for APP are not presented as there was only one farm negative to it

^c^Average lung surface affected with pneumonia (%)

Most of the pathogens co-occurred with other pathogens as shown in Table 3. The main co-infections described were farms positive to IAV, PRRSv, Mhyo (42.9 of farms) and farms positive to IAV and Mhyo (21.4%).

**Table 3:** Percentage of farms (*n* = 56) depending on co-occurrence of pathogens influenza A virus (IAV), porcine respiratory and reproductive syndrome virus (PRRSv) and *Mycoplasma hyopneumoniae* (Mhyo)^a^

IAv	PRRSv	Mhyo	Number of pathogens	Percentage (number) of farms
-	-	-	0	12.5 (7)
+	-	-	1	10.7 (6)
-	+	-	1	1.8 (1)
-	-	+	1	5.4 (3)
+	+	-	2	3.6 (2)
-	+	+	2	1.8 (1)
+	-	+	2	21.4 (12)
+	+	+	3	42.9 (24)

^a^Farm positive (+) or negative (-) to the pathogen by serology

## Discussion

This study presents detailed data on lung, heart and liver lesions in finisher pigs of a cohort of Irish farrow-to-finish farms and the first antibody prevalence report of IAV, PRRSv, Mhyo and APP across Irish farms (country level). This study relied on the Teagasc advisory services for farm selection (convenience sampling), thus, it cannot be assumed to be representative of the whole of Irish pig production. However, it represents approximately 29% of the national herd and the results obtained may provide useful insights into the prevalence of respiratory disease in Irish pig farms.

The first objective of the study was to describe the prevalence of pleurisy, pneumonia, lung abscesses, pericarditis and liver milk spots in finisher pigs of those farms. The prevalence of pleurisy is difficult to compare between countries due to the characterisation of these lesions, which is poorly described in some studies [20]. In Spain, Fraile et al. [21] presented an overall prevalence of 26.8% (cranial and dorsocaudal pleurisy), and 14.2% of dorsocaudal pleurisy, which is comparable to the 12% reported in our study. In Belgium, Meyns et al. [22] also used the SPES and reported an average pleurisy of 20.8% for scores > 1, which is comparable to the prevalence of dorsocaudal pleurisy of 13 ± 14.7% reported in the present study. Additionally, the severity of the lesions described is seldom reported. In this study, when present, lesions of dorsocaudal pleurisy were usually moderate or severe (SPES scores 3 and 4). This lesion is commonly considered to be due to infection with APP but further aetiological investigation is required to support the development and implementation of control plans on farms. The questionnaire results showed that few farmers in this study vaccinated against APP; the data on prevalence and severity of pleurisy suggest that this control measure could be of benefit on many farms. Improving the husbandry, cleaning, and disinfection, and applying strict all-in-all-out or all-forward policies for stock management are likely to prevent or attenuate the spread of APP throughout the farm [23, 24] and to decrease the prevalence of pleurisy [25].

The prevalence of pneumonia was much lower than that reported by other countries [21, 22, 26–28] but similar to the prevalence reported in Northern Ireland, UK [28]. Although this study reports an average prevalence of 13.4% of pneumonia on farms, the average surface affected of pneumonic lungs was low (6.2%). These results can be seen from two perspectives: (1) the high number of farms vaccinating for Mhyo could explain the low severity of the lesions at slaughter (only 4 out of 40 farms positive were not vaccinating); and/or (2) the onset of pneumonia caused by Mhyo is typically in the weaner stage, and most lesions heal by time of slaughter without necessarily leaving scars [27, 29, 30]. Indeed, considering that the prevalence of scars was approximately 14%, these results suggest that potentially up to almost 30% of the pigs either had pneumonia or had evidence of pneumonia (scars) over the course of their lifetime. Therefore, other methods that assess the impact of respiratory disease throughout the lifetime of the pig may be necessary to complement slaughterhouse checks. Such methods may include monitoring clinical signs (cough monitors, activity monitors) and monitoring the presence of common respiratory pathogens over time. The prevalence of abscesses was similar to that reported in the UK [28]. A limitation of the scoring method is its focus on “enzootic-pneumonia-like” lesions, partly disregarding interstitial pneumonias which have a different presentation and are notoriously difficult to score using the Madec system [10]. In Ireland, given the PRRSv and IAV prevalence nation-wide and the proportion of farms vaccinating for these diseases (at least in the study sample reported here), it would be worthwhile investigating the prevalence of viral pneumonias at slaughter.

The prevalence of pericarditis is much higher than that reported in Austria [31] and in Denmark by Nielsen et al. [32]. However, these authors maintain that the method of inspection, which avoids heart incisions, probably contributed to a lower rate of detection of this lesion. Finally, the prevalence of liver milk spots was unexpectedly high, contrasting to the much lower prevalence stated in other countries [33–35]. This suggests that stricter control programmes should be implemented on-farms.

The second objective of this study was to describe the seroprevalence of four major pathogens: IAV, PRRSv, Mhyo and APP in Irish farrow-to-finish pig farms. To the authors’ knowledge, this is the first time this information has been generated for Ireland. In our sample, herd-level prevalence was 79% for IAV, 50% for PRRSv, 71% for Mhyo, and 98% for APP. These results are comparable to those of four cross-sectional studies on respiratory disease in Spain, Belgium and France [21, 22, 26, 36]. The herd-level prevalence of IAV in the study farms is not high, considering that the ELISA kit used does not distinguish between subtypes. Fraile et al. [21] and Meyns et al. [22], tested for antibodies against IAV H1N1, H1N2, and H3N2 and concluded that over 90% of the herds were positive to those subtypes in Spain and in Belgium. Fablet et al. [36] reported a prevalence of 60 and 57.6% for subtypes H1N1 and H1N2 in French herds. Regarding PRRSv, the herd-level prevalence was similar to that reported by the French study [36], while studies in Spain (100% [26] and 89% [21]), and Belgium (88% [22]) reported a higher prevalence. The herd-level prevalence of Mhyo was similar to the prevalence reported by Fraile et al. [21], although lower when compared to the studies from Belgium and France [36, 37]. The APP herd-level prevalence is similar to that described in other studies for ApxIV detection by ELISA [13, 21, 22], and by PCR in 50 herds from Ontario, Canada [38]. Although our results indicate that virtually all farms contain pigs that are antibody positive to APP, the test does not differentiate antibodies against highly virulent serotypes from antibodies against mild serotypes. Indeed, Chiers et al. [39] stress that this serological assay cannot be used to detect subclinical infections. Thus, the clinical presentation on-farm and its relationship with pleurisy lesions at slaughter are necessary to recognise a problem [40]. As this study was designed to detect disease at farm level, the results presented on the within-farm prevalence for each disease are not discussed. Nevertheless, the authors believe that Fig. 2 offers an interesting perspective on the within-herd disease prevalence for the four pathogens studied.

Serological tests of herds have some limitations, especially in vaccinated herds. These herds will be positive by serology whether the vaccine is effective, and whether the disease is under control or not. In this study (with the exception of APP, whose particularities were discussed before), most of the herds not vaccinating for a pathogen were negative in the serology test. Likewise, the interpretation of serological results in herds in which pigs are vaccinated needs to be done carefully; results of other diagnostic tests such as pathogen detection by PCR, histopathology or immunohistochemistry are required to establish true infection and disease status.

Finally, a third objective was to analyse the associations between pluck lesions and farm health status described by serology. In the univariate approach, most of the pathogens showed associations with pleurisy and pneumonia lesions. This was probably a consequence of the high level of co-infection in pig farms as shown in Table 3. Multivariable analysis allowed removal of confounding effects and IAV was the main pathogen associated with pleurisy lesions. Although APP was probably the main etiologic factor for pleurisy [22], no farms were negative to APP and its effect on pleurisy lesions could not be studied. Thus, whether influenza alone was associated with pleurisy or it acted as a triggering or aggravating factor cannot be elucidated. However, it is known that viral infections can favour the effects of bacterial pathogens like APP or *Pasteurella multocida* in pigs [1]. As expected, Mhyo was the main pathogen associated with pneumonia and scars. Mhyo is well known to be the main etiologic agent in pneumonia [8]. However, the multivariable analysis also showed some association of IAV in the pneumonia lesions. The importance of this co-infection in the development of pneumonia should be further studied [41]. Pericarditis also showed an association with positive status for Mhyo. This is an interesting result in light of a 1997 study which reported the microbiological and pathological findings of 46 cases of chronic pericarditis in Danish finisher pigs at slaughter [42]. In this study, Buttenschon et al. found that Mhyo was involved in 33 out the 46 cases reported. However, the chronic character of the lesions assessed at slaughter dictates uncertainty when attributing a cause-effect which links pathogens to lesions. Other pathogens like *Glaesserella parasuis* and *Streptococcus suis* have been associated with pericarditis and further studies should investigate this association.

## Conclusions

Most of the farms affected with pleurisy had moderate to severe lesions. On the contrary, most of the farms affected with pneumonia had mild lesions, which could be the effect of the generalised use of Mhyo vaccination in piglets. At the same time, the prevalence of liver milk spots was unexpectedly high which denotes the need for stricter control programmes on-farm. The herd-level prevalence of IAV, PRRSv, Mhyo and APP in the present study sample is similar to or lower than in other European countries. Pleurisy levels were higher in those herds positive to IAV and pneumonia was associated with positive status for Mhyo and IAV. Further research on the PRRSv and APP strains circulating in Ireland are necessary to support the design of national or regional control plans.

## Data Availability

The datasets used and/or analysed during the current study are available from the corresponding author on reasonable request.

## Abbreviations

APP: *Actinobacillus pleuropneumoniae*
ePM: e-Profit Monitor
IAV: Influenza A virus
Mhyo: *Mycoplasma hyopneumoniae*
PRRSv: Porcine reproductive and respiratory syndrome virus
